# Pathogenesis, clinical features, and treatment of plurihormonal pituitary adenoma

**DOI:** 10.3389/fnins.2023.1323883

**Published:** 2024-01-08

**Authors:** Yunjia Cai, Siyuan Liu, Xue Zhao, Linan Ren, Xinming Liu, Xiaokun Gang, Guixia Wang

**Affiliations:** Department of Endocrinology and Metabolism, The First Hospital of Jilin University, Changchun, China

**Keywords:** pituitary adenoma, plurihormonal, stem cell, immunohistochemistry, pathogenesis

## Abstract

Plurihormonal pituitary adenoma (PPA) is a type of pituitary tumor capable of producing two or more hormones and usually presents as an aggressive, large adenoma. As yet, its pathogenesis remains unclear. This is the first study to systematically summarize the underlying pathogenesis of PPA. The pathogenesis is related to plurihormonal primordial stem cells, co-transcription factors, hormone co-expression, differential gene expression, and cell transdifferentiation. We conducted a literature review of PPA and analyzed its clinical characteristics. We found that the average age of patients with PPA was approximately 40 years, and most showed only one clinical symptom. The most common manifestation was acromegaly. Currently, PPA is treated with surgical resection. However, recent studies suggest that immunotherapy may be a potentially effective treatment.

## Introduction

Pituitary neuroendocrine tumors, formerly known as pituitary adenomas, are usually benign monoclonal tumors (Neou et al., 2020; Cui et al., 2021). The incidence is approximately 0.07–0.15% and accounts for 15% of all intracranial tumors (Ho et al., 2001; Fernandez et al., 2010; Raappana et al., 2010; Mete and Lopes, 2017). Most pituitary adenomas produce only one hormone, and the production of multiple hormones is rare (Daly et al., 2006; Ostrom et al., 2014; Melmed, 2020; Asa et al., 2022; Graffeo et al., 2022; Yao et al., 2022). Plurihormonal pituitary adenoma (PPA) is defined as the immunopositivity of two or more hormones within the same cell (haplomorphism), or two or more distinct cell populations with different hormones (pleomorphism) (Thapar et al., 1993; Ho et al., 2001; Liu et al., 2018; Asa et al., 2022). Initially thought to be an atypical variant, an increasing number of immunohistochemical, ultrastructural, and biochemical analyses have confirmed PPA as a type of pituitary tumor. According to the 2017 classification of pituitary tumors by the World Health Organization (WHO), PPA is divided into two subtypes: pituitary transcription factor 1 (PIT-1)-positive plurihormonal pituitary adenomas (PIT-1 + PPA) and plurihormonal adenomas with unusual immunohistochemical combinations (PAwUIC). Notably, WHO suggested that growth hormone-prolactin (GH-PRL) and follicle-stimulating hormone-luteinizing hormone (FSH-LH) adenomas are not PPA (Rasul et al., 2014; Villa et al., 2014; Lopes, 2017; Mete and Lopes, 2017; Nf and Ai, 2023).

Plurihormonal pituitary adenomas were previously considered rare. Recent application of immunohistochemistry to pituitary tumors has shown that many pituitary adenomas, including clinically nonfunctional pituitary adenomas, are actually plurihormonal (Salehi et al., 2006; Chinezu et al., 2017; Kobalka et al., 2021). Recent studies have also found that PPA accounts for 10–15% of all pituitary tumors (Shi et al., 2022). PPA can be more aggressive than pituitary tumors that secrete a single hormone, with more than 50% of the tumors being locally aggressive (Pawlikowski et al., 2010; Andrews et al., 2021). PPA is usually a large adenoma with a high recurrence rate (Pawlikowski et al., 2010). Marek et al. analyzed 155 excised pituitary adenomas and found that more than one-third of the pituitary adenomas were pluripotent, and that the recurrence rate of plurihormonal adenomas was twice that of single-hormone adenomas (17.8% vs. 9.4%). They also found that adrenocorticotropic hormone (ACTH)-expressing adenomas in plurihormonal nonfunctional pituitary tumors had a higher recurrence rate than ACTH-negative adenomas (35.3% vs. 14.2%) (Pawlikowski et al., 2010). Furthermore, several studies have also shown that the Ki-67 index of PPA is significantly higher than that of single-hormone adenomas (Thapar et al., 1993; Pawlikowski et al., 2010; Luk et al., 2012).

Ultrastructural studies have shown that PPA can be classified as monomorphic and pleomorphic adenomas. Monomorphic adenomas consist of single cells capable of producing two or more hormones. Pleomorphic adenomas are composed of a variety of cell types, and different cells produce different hormones. The immunoelectron microscopy colloidal gold double-labeling technique has shown that multiple hormonal profiles can co-exist within a single cell, even in pleomorphic adenomas. Chiang et al. immunostained 167 cases of PPA and observed the presence of multiple hormone mixtures in individual adenoma cells. Therefore, they concluded that most PPAs are probably monomorphic adenomas (Ho et al., 2001).

In 2022, the WHO proposed that the adenohypophyseal is composed of at least six cell types: somatotrophs, lactotrophs, mammosomatotrophs, and thyrotrophs of the PIT1 lineage; corticotrophs of the T-box family member TBX19 (T-PIT) lineage; and gonadotrophs of the steroidogenic factor-1 (SF1) lineage (Mariniello et al., 2019; Alatzoglou et al., 2020; Asa et al., 2021; Kobalka et al., 2021; Asa et al., 2022; Villa et al., 2023). Studies have shown that the pathogenesis of pituitary adenoma may be related to gene mutations, chromosome number variations, DNA methylation, microRNA regulation, and transcription factor regulation (Asa and Ezzat, 2009; Salomon et al., 2018; Vandeva et al., 2019; Chang et al., 2020; Sabatino et al., 2022). However, the pathogenesis of PPA is still unclear. This study aimed to describe the potential pathogenesis of PPA and analyze its clinical characteristics.

## Potential mechanisms

The pathogenesis of PPA is unclear (Srirangam Nadhamuni and Korbonits, 2020). The underlying mechanism may be as follows.

### Plurihormonal primordial stem cell

The anterior pituitary gland originates from the Rathke sac. Stem cells in the anterior pituitary gland proliferate in the embryo, and stem cells in the embryo differentiate into different pituitary cells under the regulation of various transcription factors (Mete et al., 2018; Alatzoglou et al., 2020; Matsumoto et al., 2020; Zhang et al., 2020; Matsumoto and Takahashi, 2021). Over the past decade, evidence for the existence of adult pituitary stem cells (PSC) has been provided using *in vitro* clonogenic assays, flow cytometry side-population analyses, immunohistochemical analyses, and genetic methods. These cells can self-regenerate, and undergo pluripotent differentiation to produce all the hormone lineages in the anterior pituitary (Carreno et al., 2017; Cox et al., 2017; Haston et al., 2018; Tordjman et al., 2019; Laporte et al., 2020; Würth et al., 2020; Willis et al., 2022; Winningham and Camper, 2023). PSC is thought to be present in the intermediate lobe (IL) and the dorsal anterior lobe (AL), in the marginal zone (MZ), and is dispersed throughout the AL parenchyma (Garcia-Lavandeira et al., 2012; Haston et al., 2018). Garcia et al. found that cell populations located in MZ co-express GFRα2, RET, and PROP1, and that these cells contribute to the stem cell niche in MZ and may play a role in regulating structural guidance and the survival of PSC (Garcia-Lavandeira et al., 2009). Chen et al. found that PSC expressed SOX2, SOX9, CD4, CD133, and SCA1 (Chen et al., 2005). Previous *in vitro* clonal assay studies have demonstrated the expression of PROP1, PRX1/2, GFRα2, CXCR4, and NESTIN in PSC (Horiguchi et al., 2012; Rizzoti et al., 2013; Higuchi et al., 2014). Notably, cancer cells with stem cell properties are called cancer stem cells (CSC), and these cells can self-regenerate and differentiate into all cell types within the tumor (Batlle and Clevers, 2017; Steinbichler et al., 2018; Lan and Behrens, 2023). More recent studies have identified these cells in pituitary adenomas (Carreno et al., 2017; Cox et al., 2017; Lodge et al., 2019).

Xu et al. first discovered the presence of stem cells in pituitary adenomas that expressed pituitary-specific markers, such as PIT-1 and stem cell markers OCT4, NOTCH4, CD133, and NESTIN (Xu M. et al., 2009). Wurth et al. isolated CSC-like cell populations from 38 human pituitary adenomas that expressed CD133, OCT4, SOX2, and NESTIN, with the potential to differentiate into hormone-producing cells (Würth et al., 2017). These findings support a potential role for adult pituitary stem cells in pituitary adenomas. Almeida et al. suggested that the occurrence of plurihormonal and null cell-type adenomas (no distinguishable ultrastructural, immunohistochemical, or histologic characteristics of recognized adenohypophyseal cell types) supports the hypothesis that pituitary stem cells may be a potential source of pituitary adenomas (De Ameida et al., 2010). Radian et al. found that GH-and thyroid-stimulating hormone (TSH)-secreting cells share common progenitor cell expression. This suggests that TSH adenomas originate from pituitary stem cells that differentiate into GH, PRL, or TSH cells, which may play a role in the pathogenesis of TSH-secreting PPA (Teramoto et al., 2004; Vankelecom and Roose, 2017). Matsuno et al. studied the expression of pituitary hormones including glycoprotein hormone mRNA in GH and PRL adenomas using non-isotope *in situ* hybridization. They found that some cells in GH adenomas expressed multiple hormone genes, mainly ACTH, FSH, and LH. These results suggest that some GH adenomas may originate from adult pituitary stem cells, which are precursors of various hormone-expressing cells (Matsuno et al., 1995, 1996). For example, Kovacs et al. reported a case of PPA-producing GH and ACTH. Through immunohistochemical analysis, fluoroscopic electron microscopy, immunoelectron microscopy, and *in situ* hybridization, the tumor was found to be composed of two independent cell types. Moreover, one cell population synthesized GH, and the other synthesized ACTH. The authors proposed that PPA may originate from undifferentiated stem cells that can differentiate into many different cell types and that this multidirectional differentiation could explain the pathogenesis of PPA (Kovacs et al., 1998).

**Figure 1:**
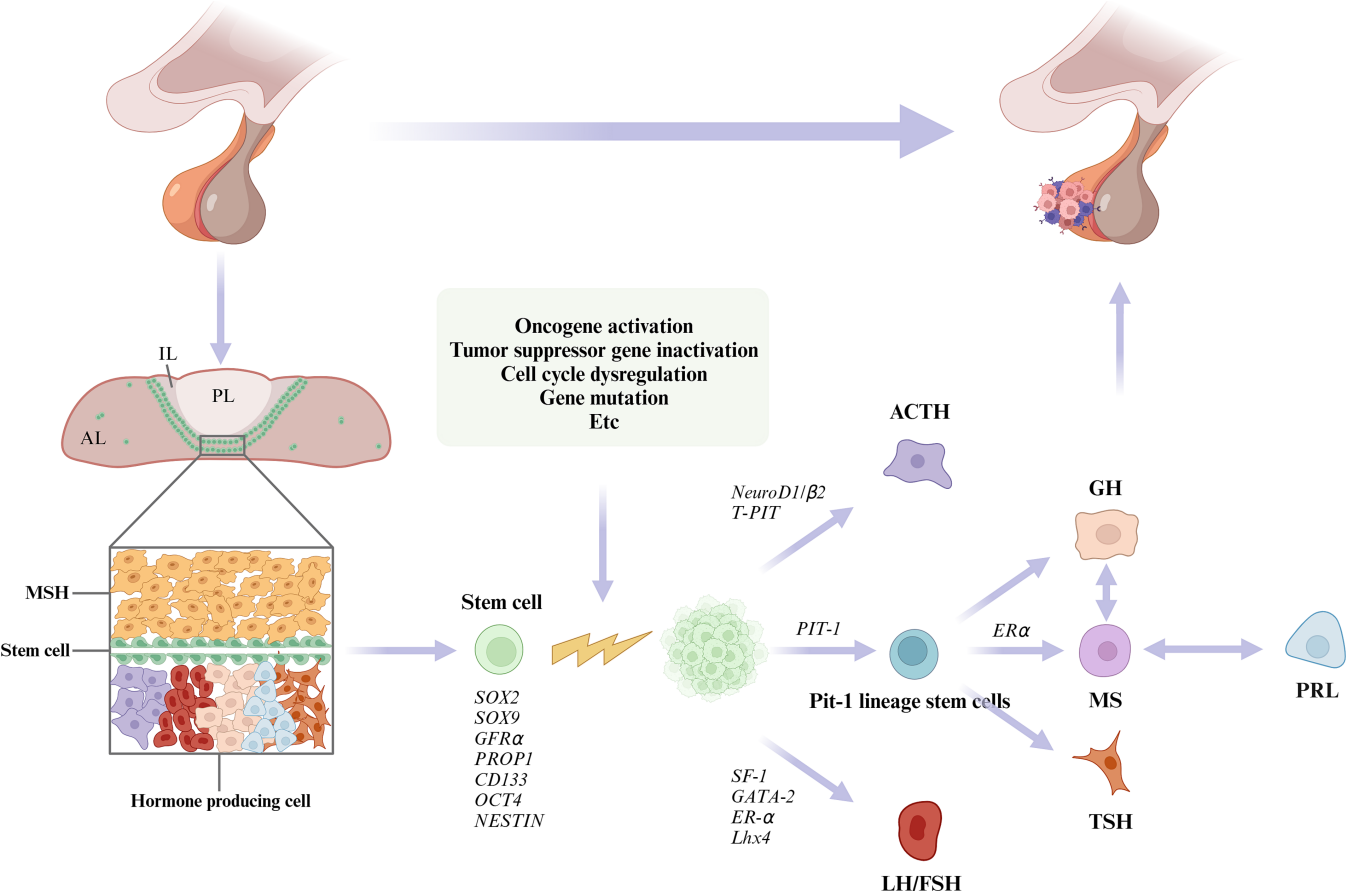
The pituitary gland consists of the anterior lobe (AL), intermediate lobe (IL), and posterior lobe (PL). Plurihormonal primordial stem cells (PSC) are thought to reside in the intermediate lobe (IL) and the dorsal anterior lobe (AL), and are dispersed throughout the AL parenchyma. PSCs proliferate into adenomas under conditions that activate oncogenes, inactive tumor suppressor genes, dysregulate cell cycles, or cause mutations. Under the regulation of different transcription factors, the PSCs differentiate into different cells and secrete a variety of hormones.

**Figure 2:**
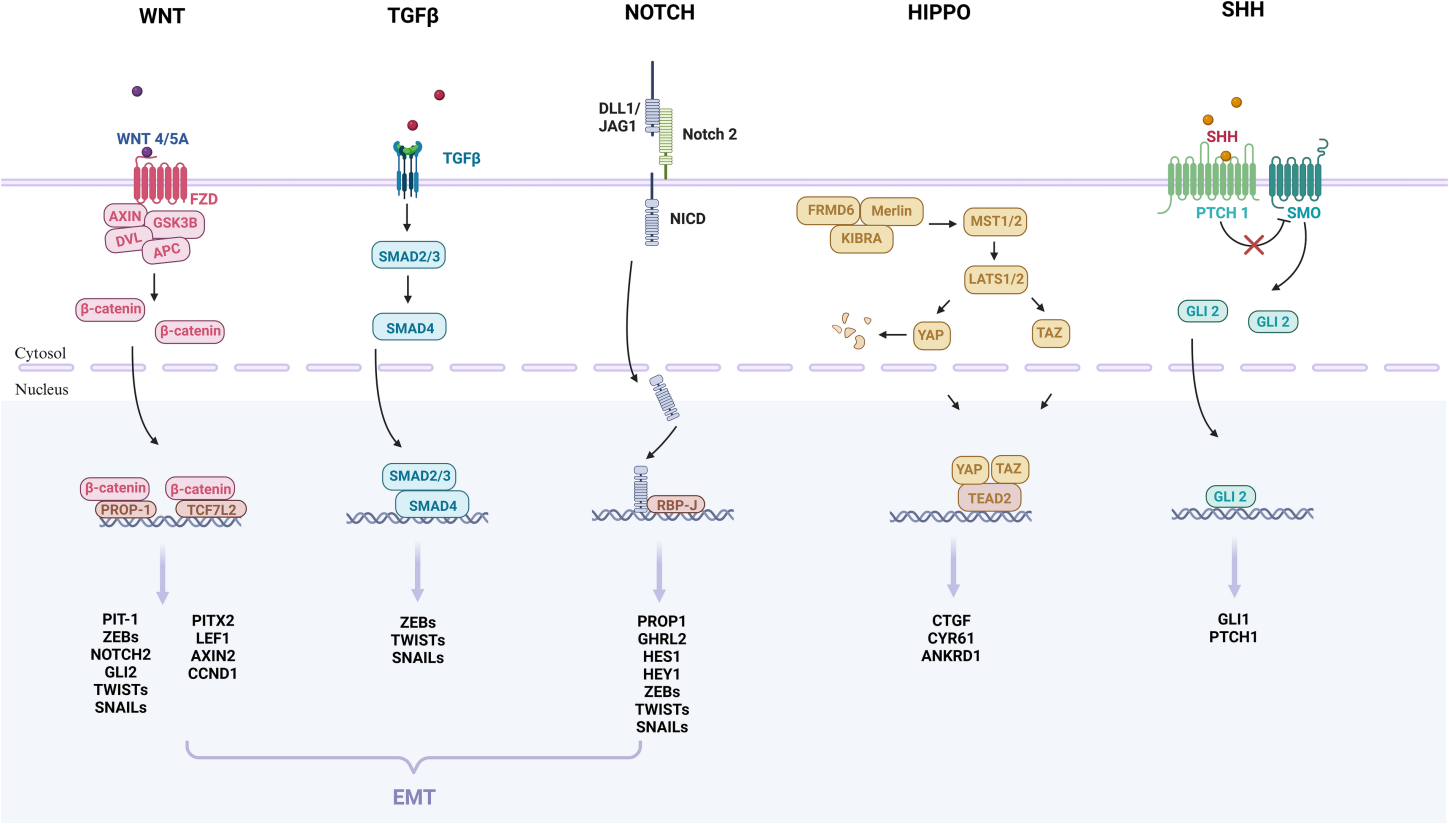
These are the key signaling pathways of pituitary adenomas that may be involved in the initiation and progression of adult pituitary stem cell tumors. The pathways include WNT, TGFβ, NOTCH, HIPPO and SHH. The WNT, TGFβ, and NOTCH pathways may further participate in epithelium-mesenchymal transition (EMT), and promote tumor growth and spread. The WNT pathway: The WNT protein binds to the FZD receptor on the cell membrane, leading to the stabilization and accumulation of β-catenin protein, which enters the nucleus and binds to the transcription factor PROP-1, TGF7L2, regulating the expression of the target gene. The TGFβ pathway: TGFβ binds to the receptor complex, phosphorylates to SMAD2/3, and binds to SMAD4 to form a complex that enters the nucleus and binds to transcription factors to regulate the expression of target genes. The NOTCH pathway: After the NOTCH receptor binds to ligand DLL1/JAG1, NICD is released, which enters the nucleus and binds to transcription factor RBP-J to regulate gene expression. The HIPPO pathway: The protein encoded by the Hippo gene interacts with MST to activate the LATS protein, so that YAP and TAZ are phosphorylated and fixed in the nucleus, unable to enter the nucleus to regulate gene expression. When the signaling pathway is suppressed, YAP and TAZ enter the nucleus, bind to the transcription factor TEAD, and regulate gene expression. SHH pathway: In the absence of SHH, the PTCH receptor inhibits the activity of the protein SMO, and when SHH binds to PTCH, PTCH no longer inhibits SMO, thus SMO is activated and GLI protein is regulated. These proteins enter the nucleus to regulate the expression of target genes. EMT: Three families of transcription factors, ZEB, SNAIL, and TWIST, play a central role in EMT. For pituitary stem cell tumors, WNT, TGFβ, and NOTCH pathways may be involved in the regulation of EMT.

In this study, we summarized the underlying pathogenesis (Figure 1). A growing number of studies have identified the presence of PSC in the pituitary gland. PSC can grow into adenomas under conditions such as activation of oncogenes and inactivation of oncogenes (Carreno et al., 2017; Laporte et al., 2020; Würth et al., 2020; Winningham and Camper, 2023). Under the regulation of different transcription factors, they differentiate into different cells and secrete different hormones, which ultimately lead to PPA. In addition, we found that the WNT, TGFβ, NOTCH, HIPPO and SHH pathways may be associated with adult pituitary stem cell tumorigenesis (Gaston-Massuet et al., 2011; Lodge et al., 2019; Würth et al., 2020; Winningham and Camper, 2023). (Figure 2) Further basic research on adult pituitary stem cells is needed to explore their role in the development, progression, and recurrence of PPA.

### Co-transcription factor

Previous studies identified several transcription factors that regulate the transformation of cellular precursors into mature secretory cells, including T-pit, Pitx1, Pitx2, Prop-1, Pit-1, SF-1, NeuroD1, and GATA-2 (Parvin et al., 2017; Sjöstedt et al., 2017; Ellsworth and Stallings, 2018; Mete et al., 2018; Cheung and Camper, 2020; Hickman et al., 2021). Pitx1 is a universal transcription factor found in all types of pituitary cells and all types of pituitary adenomas. Pitx1 acts synergistically with NeuroD1/2, SF-1, and Pit-1 to activate POMC/ACTH, FSH-LH, and GH-PRL-TSH transcription, respectively (Szeto et al., 1999; Lamolet et al., 2001). Matsuno et al. described a GH-PRL-ACTH pituitary tumor and demonstrated the use of immunohistochemical staining, *in situ* hybridization, and cell culture techniques to generate GH, PRL, ACTH, and Pit-1 expression. They believed that in addition to Pit-1, there may be unknown transcription factors that simultaneously express GH, PRL, and ACTH (Matsuno et al., 1996). Shigeyuki et al. found that GH and ACTH are expressed in the same cell in one GH-ACTH adenoma, suggesting that GH is produced in ACTH-derived adenoma cells and that Pit-1 and NeuroD1 are expressed in the nucleus of the same adenoma cells. This suggests that the production of multiple cell lineage hormones in the same cell may be due to abnormal expression patterns associated with the transcription factors in pituitary tumors. The synergistic mechanism of NeuroD1 and Pit-1 is of great scientific significance and requires further investigation (Tahara et al., 2002).

### Co-expression of hormones

Normally, a cell synthesizes and secretes specific hormones for the regulation of physiological functions and metabolic processes. However, some normal cells and adenoma cells express and secrete many different types of hormones, a phenomenon known as “hormone co-expression” (Radian et al., 2003; Tong et al., 2013; Allensworth-James et al., 2020). Osamura et al. observed the co-expression of GH, α subunits, and gonadotropin in some cells of the anterior pituitary in normal adults (Osamura et al., 2009). This evidence suggests the presence of a certain percentage of plurihormonal cells in the normal anterior pituitary, which refutes the notion of “one cell type, one hormone.” Lubov et al. found that the same cells in normal adenohypophysis could co-express PRL with ACTH, TSH, FSH, and LH; GH with ACTH, TSH, FSH, and LH; and TSH with ACTH, FSH, and LH. After comparing 10 scanning chemical sections of the pituitary gland, they found a significant overlap in the GH, PRL, LH, and ACTH expression regions of the distal pituitary gland. Moreover, the co-expression of TSH and PRL was highly prominent in 50% of the anterior pituitary cells using confocal scanning microscopy, with co-expression coefficients ranging from 3.4 to 22%. The co-expression coefficients of PRL and LH were between 4 to15%. The coexpression coefficients of GH and LH ranged from 0.4 to 24%. The coexpression coefficients of ACTH/PRL and ACTH/GH were 34 and 55%, respectively (Mitrofanova et al., 2017). Therefore, this hormone co-expression phenomenon may also be the cause of PPA. Evidence from immunohistochemistry studies has shown that GH and PRL are co-expressed in the same cell (Kurotani et al., 2001; Tong et al., 2013). Likewise, the cells in pituitary adenomas may display hormone co-expression. Electron microscopy, immunocytochemistry, and laser scanning confocal microscopy revealed that GH and TSH were co-expressed in the anterior pituitary cells of adult rats with methimazole-induced hypothyroidism (Radian et al., 2003). Tong et al. performed immunohistochemical tests on a patient with TSH-GH-PRL multihormone adenoma and found that TSH and GH were colocalized in most tumor cells, indicating that the two hormones were mainly expressed by the same cells. The ultrasonic structure presented a simplex cell appearance and a single type of secretory particle, similar to the secretory particles in normal TSH cells. This suggests that TSH and GH originated from the same secretory particles (Tong et al., 2013).

### Differential gene expression

PPA pathogenesis may involve several abnormally expressed genes and pathways. Zhiquan et al. examined the gene expression profiles of seven PPA using a magnetic bead-based optical fiber array, and compared them with those of three normal pituitary glands. A pathway analysis of all differentially expressed genes using the Kyoto Encyclopedia of Genes and Genomes (Jiang et al., 2012) demonstrated that the expression of six genes in PPA increased significantly, and the expression of 334 genes and 15 expression sequence tags decreased significantly. Bioinformatics analysis indicated that HIGD1B, EPS8, ECT2, and BTG2 may play important roles in the tumorigenesis and progression of PPA. Pathway analysis showed that the Notch and p53 signaling pathways may play an important role in the development of PPA tumors. Extracellular matrix (ECM) receptor interactions may play a role in inhibiting tumor invasion and metastasis (Table 1).

**Table 1:** Genes and pathways that may be involved in abnormal expression in PPA.

Differentially expressed genes in PPA	Signaling pathway	Function	Expression in PPA	Potential role in PPA	References
HIGD1B	NA	Hypoxia induced the expression of HIG1 and HIG2	Overexpressed	May increase tumor hypoxia tolerance or promote angiogenesis and drug resistance	Jiang et al. (2012), Denko et al. (2000)
EPS8	NA	Amplify growth factor receptor signaling in pituitary tumors to promote proliferation and cell survival	Overexpressed	May promote tumor proliferation and invasion	Jiang et al. (2012), Xu Q. et al. (2009)
ECT2	NA	An important link between the cell-cycle machinery and Rho signaling pathways that are involved in the regulation of cell division	Overexpressed	May promote tumor proliferation and invasion	Jiang et al. (2012), Tatsumoto et al. (1999)
BTG2	NA	An antiproliferative tumor suppressor gene, as its overexpression leads to arrest of cells at the G1 phase of the cell cycle	Underexpressed	May promote tumor proliferation	Jiang et al. (2012), Rouault et al. (1996)
COL11A2, ITGB4, THBS4, COL4A6, COL11A1, COL6A2, COL6A1	ECM-receptor Interactions	It has a structural support function as well as a role in Cell adhesion, migration, proliferation, and survival	Underexpressed	May inhibit tumor invasion and metastasis	Jiang et al. (2012), Paez-Pereda et al. (2005)
DLK1	Notch	As a growth factor, maintaining proliferating cells in an undifferentiated state	Underexpressed	May promote the directed differentiation of secretory cells	Jiang et al. (2012), Yevtodiyenko and Schmidt, 2006, Nakakura et al. (2009)
CtBP2	Notch	Act in the nucleus as transcriptional corepressors and in the cytoplasm as regulators of Golgi apparatus fission	Underexpressed	May inhibit tumor invasion and malignant transformation	Jiang et al. (2012), Bergman et al. (2009)
HES1, HES5	Notch	Are essential Notch effectors in regulation of mammalian neuronal differentiation	Underexpressed	May be related to cell differentiation and inhibition of excessive tumor growth	Jiang et al. (2012), Ohtsuka et al. (1999)
Ep300	Notch	Controls enhancer acetylation by interacting with TFAP2β	Underexpressed	NA	Jiang et al. (2012), Durbin et al. (2022)
GADD45	p53	Can inhibit cell proliferation at different stages, including the G1-S and G2-M checkpoints, and induce cell apoptosis	Underexpressed	May promote tumor proliferation and progression	Jiang et al. (2012), Ying et al. (2005)
Reprimo	p53	Induces cell-cycle arrest by inhibiting Cdc2 activity and nuclear translocation of the Cdc2/cyclin B1 complex	Underexpressed		Jiang et al. (2012), Li et al. (2022)
IGFBP3	p53	By blocking IGF-1 survival signaling to the IGF-1 receptor	Underexpressed		Jiang et al. (2012), D'addio et al. (2022)
PMAIP1	p53	An important mediator of p53-related apoptosis	Underexpressed		Jiang et al. (2012), Wang et al. (2021)

EPS8, epidermal growth factor receptor pathway substrate 8; ECT2, epithelial cell transforming sequence 2; BTG2, B cell translocation gene 2; COL11A2, alpha2 chain of type XI collagen; ITGB4, integrin beta4; THBS4, thrombospondin 4; COL4A6, alpha 6 chain of type IV collagen; COL11A1, alpha1 chain of type XI collagen; COL6A2, alpha2 chain of type IV collagen; COL6A1, alpha1 chain of type IV collagen; DLK1, the Notch1 inhibitor delta-like 1 homolog; CtBP2, transcriptional corepressor C-terminal binding protein 2; HES1, the hairy enhancer of the split 1; Ep300, histone acetyltransferase p300; GADD45, growth arrest and DNA damage-inducible gene 45; IGFBP3, insulin-like growth factor binding protein 3; PMAIP1, phorbol-12-myristate-13-acetate-induced protein 1.

### Cell transdifferentiation

In the past few decades, the “one cell, one hormone” theory has been widely accepted, and it has been proposed that the phenotype of adenopituitary cells is immutable, and that they are irreversibly committed to producing a single hormone (Kovacs et al., 1989). In recent years, new technologies have emerged to disprove this. Evidence has shown that polyhormone cells may exist in some differentiated cellular pathways (Stefaneanu et al., 1992; Mitrofanova et al., 2017; Asa et al., 2023). Normal gonadotrophs produce gonadotropins, FSH, and LH. Mammosomatotrophs are cells that produce both GH and PRL, and represent a fluid cell type that can transdifferentiate into somatotrophs during growth and lactotrophs during pregnancy (Stefaneanu et al., 1992; Asa et al., 2023). Childs et al. found that a single cell of the pituitary gland in rodents can contain both ACTH and FSH, and explained this observation as “transdifferentiation.” One cell type may be transformed into another owing to the genetic and epigenetic factors at play during tumor progression, and are often accompanied by different morphological changes (Vidal et al., 2000). Kovacs et al. reported a case of an ACTH-GH pituitary tumor and identified ACTH in several GH-secreting particles using immunoelectron microscopy. This result suggests that the tumor was originally a GH adenoma that started producing ACTH because of mutations that occurred during tumor progression (Kovacs et al., 1998). Mark et al. reported a case of adenohypophyseal hyperplasia in a patient with primary hypothyroidism, in which TSH and PRL cells proliferated, and GH cells transdifferentiated into cells that secreted both GH and TSH (Jentoft et al., 2012). Sergio et al. found GH-and TSH-labeled double-hormone particles in the cells of TSH-GH adenomas and double-hormone particles in the adenomas of two patients with acromegaly labeled with PRL and TSH, respectively. Using double immunogold labeling in GH-PRL adenomas, they observed that most tumor cells contained dual hormone particles, and some cells contained only GH or PRL particles. These findings strongly suggest that the transdifferentiation of one cell type into another may be the cause of PPA (Vidal et al., 1999). It has been suggested that the interconversion of one cell type into another is not a direct process but occurs through a bihormonal transition cell that contains functional components common to both cells (Frawley and Boockfor, 1991).

### Clinical features

Studies have found that approximately 70% of patients with PPA have hormonal symptoms, with acromegaly being the most common (accounting for 50% of cases). Hyperprolactinemia-related symptoms account for 20% of all the cases, whereas gonadotropin-and thyrotropin-related symptoms are rare (Ho et al., 2001).

Ruoyu et al. analyzed the clinical characteristics of 70 patients with PPA and found that these adenomas accounted for 13% of all pituitary adenomas recorded during the same period. 53% of the patients had one clinical symptom, 1% had two endocrine symptoms, and none of the patients showed three or more endocrine symptoms. Of the patients, 74.3% had an elevated level of one hormone, 7.1% had an elevated level of two hormones, and only one had an elevated level of all three hormones (Shi et al., 2022). Seckin et al. analyzed the clinical features of 27 patients with PPA, 10 of whom were diagnosed with PAwUIC and 9 with PIT-1 + PPA. The median age was 44.7 years (range, 19–75 years) and 59.3% were males and 40.7% were females. Large adenomas accounted for 88.8%, giant adenomas ≥4 cm accounted for 22.2, 44.4% of patients were accompanied by cavernous sinus infiltration, and nearly half of the patients were highly invasive. 77.8 of the patients with PAwUIC, 77.8% showed non-functional pituitary adenomas, and only four patients had hormonal secretory characteristics (Aydin et al., 2019). Mete et al. reported 31 patients with monomorphic multihormone Pit-1 lineage tumors with a mean age of 44.3 years, 11 of whom had hormone secretion symptoms. Most patients have large adenomas, and 30% have tumors that invade the cavernous sinus (Mete et al., 2016). Horvath et al. reported 29 multihormone Pit-1 spectrum tumors in patients with a mean age of 37.5 years, predominantly female (25:4), all of which were macroadenomas, and approximately half of the tumors were aggressive (Horvath et al., 2005). Erickson et al. reported 27 patients with plurihormonal Pit-1 spectrum tumors (mean age, 36.4 years). All tumors were macroadenomas, and 60% of the tumors were aggressive (Erickson et al., 2009). Sylvia et al. reported that PPA co-expressed with Pit-1 and SF-1 in 38 patients (mean age, 53 years; range, 12–79 years). Most tumors had lamellar or nested adherent epithelioid cells that were eosinophilic throughout the intestine. The most commonly secreted hormone is the growth hormone (GH), which causes acromegaly. However, central hyperthyroidism is rare. Furthermore, GATA3 expression was detected in a subset of PPA that expresses both Pit-1 and SF-1 (Asa et al., 2023).

Although PPA immunohistochemistry usually shows a positive expression of multiple hormones, it does not clinically show an increased secretion of multiple hormones. Most patients only show one clinical symptom (Ho et al., 2001), We analyze the possible reasons as follows. (1) Some immunohistochemically positive hormones have fewer positive particles and the hormone content is insufficient (Elhadd et al., 2009; Vora and Karunakaran, 2017). (2) Although tumor cells can synthesize certain hormones, the secretion mechanism may be obstructed, and when the secretion of one hormone is dominant, the secretion of another may be suppressed (Lamberts and Macleod, 1990; Müller et al., 1999). (3) Tumors may originate from primitive cells that are not biologically active. Tumor cells can only synthesize hormonal precursors. Although they can cross-react with the corresponding antibody and appear immunopositive, they do not exhibit biological activity (Tordjman et al., 2019).

We have summarized 31 cases of PPA reported in the literature (Table 2). Most patients had aggressive macroadenomas, and the mean age of the patients was 46.51 years, ranging from 13 to 72 years, with 42% men and 58% women. The most common symptom was acromegaly, and most patients opt for surgical tumor removal.

**Table 2:** Published reports on plurihormonal pituitary adenomas.

IHC	Sex/Age	Size (cm)	Location	Aggressive	Manifestation	Elevate hormonal values	Therapy	Recovery	References
FSH-GH-LH-TSH-PRL	F/65	2.2*1.8*2.3	Intrasellar	NA	Headache	None	SURGERY	Recovery	Al-Shraim et al. (2009)
PRL-GH-TSH-LH-FSH	M/66	0.3*0.25	Anterior pituitary	NA	Mental derangement	NA	NA	Death	Tomita et al. (1981)
TSH-GH-ACTH-LH-FSH	F/40	NA	Suprasellar extension and compression of the optic chiasma	Yes	Thyrotoxicosis and acromegaly	GH, ACTH, LH, FSH	Surgery	NA	Maisnam et al. (2012)
TSH-GH-LH-PRL	F/60	2.3*2.2*2.0	Intrasellar deviation to the right	NA	Thyrotoxicosis, headache and blurred vision	TSH	Surgery	Recovery	Luk et al. (2012)
GH-TSH-ACTH-PRL	M/64	3.0*2.4*1.9	Surrounding the right internal carotid artery, suprasellar extension and compression of the optic chiasma	Yes	Thyrotoxicosis	TSH, PRL	Surgery	Recovery	Vora & Karunakaran, (2017)
TSH-GH-PRL-ACTH	F/42	2.3*2.0	Located in the left intrasellar and suprasellar regions, invading the cavernous sinus and extending to the skull base	Yes	Thyrotoxicosis	TSH, GH, PRL	Medicine and Surgery	Recovery	Tordjman et al. (2019)
TSH-FSH-ACTH	M/29	4.6*3.8*3.6	Downward into the sphenoid sinus, upward into the suprasellar cistern, and outward into the left cavernous sinus	Yes	Thyrotoxicosis	TSH, FSH	Surgery	Recovery	Yoshida et al. (2008)
TSH-GH-FSH	F/67	1.5*1.3*1.1	Adjacent to the optic chiasma, extending to the right cavernous sinus	Yes	Thyrotoxicosis	TSH	Surgery	Recovery	Elhadd et al. (2009)
PRL-GH-TSH	F/12	8.0*5.0*5.5	Anterior pituitary gland and middle cranial fossa	NA	Hyperprolactinemia, acromegaly, thyrotoxicosis	PRL, GH, TSH	Medicine	Recovery	Moszczyńska et al. (2021)
GH-TSH-PRL	M/48	NA	NA	NA	Acromegaly	GH, TSH	Surgery	Recurrence	Felix et al. (1994)
FSH-LH-TSH	F/39	2.5	Tumor hemorrhage into the cavernous sinus	Yes	Hyperprolactinemia, sporadic menstruation	FSH	Surgery	Recovery	Sommergruber et al. (2000)
ACTH-GH-PRL	F/65	2.9*1.5	Invading the top of the sphenoid sinus cavernous sinus and compressing the optic chiasma	Yes	Cushing’s syndrome, headache, visual field defects	ACTH, PRL	Surgery	Recovery	Allehaibi et al. (2021)
GH-PRL-ACTH	M/27	2.8	Invading the right cavernous sinus and surrounding the right internal carotid artery	Yes	Acromegaly	GH, PRL, ACTH	Surgery	Recovery	Matsuno et al. (1996)
PRL-TSH-LH	M/23	NA	NA	NA	diminution of vision	PRL, LH	Surgery	NA	Mccomb et al. (1984)
TSH-GH-PRL	F/48	NA	NA	NA	Thyrotoxicosis and acromegaly	TSH, GH, PRL	Surgery	Recovery	Shimatsu et al. (1999)
TSH-GH-PRL	M/31	3.8*3.3*3.5	Sellar-suprasellar, encased bilateral cavernous sinuses and compressed the optic chiasm antero-superiorly	Yes	thyrotoxicosis, headache and neck swelling	TSH, GH, PRL	Surgery	Recurrence	Goh et al. (2023)
ACTH-GH-PRL	M/45	3.1*2.0*2.2	Located from inside the sella turcica to upside the sella turcica	Yes	Acromegaly	GH, ACTH	Surgery	Recurrence	Takiguchi et al. (2017)
ACTH-LH	M/72	NA	Located in the intrasellar region and extending to the suprasellar region	Yes	Headache and high blood pressure	NA	Surgery	Recurrence	Villa et al. (2014)
ACTH-LH	F/67	3.5	Invading suprasellar region, paranasal sinus and cavernous sinus	Yes	Cushing’s syndrome	ACTH	Surgery	Recovery	Egensperger et al. (2001)
GH-LH	M/40	1.0	Extending to the right cavernous sinus	Yes	Acromegaly	GH	Surgery	Recovery	Salehi et al. (2006)
LH-PRL	F/30	2.0	Intrasellar	NA	Amenorrhea, infertility and hyperprolactinemia	LH, PRL	Medicine and Surgery	NA	Saveanu et al. (2001)
TSH-GH	M/38	2.7	Located in the intrasellar region and extending to the suprasellar region	NA	Thyrotoxicosis and acromegaly	TSH, GH	Surgery	NA	Kuzuya et al. (1990)
ACTH-GH	F/53	NA	Left lobe of pituitary gland	NA	Cushing’s syndrome	ACTH	Surgery	NA	Tahara et al. (2002)
ACTH-GH	M/36	NA	Left lobe of pituitary gland	NA	Acromegaly	GH, ACTH	Surgery	NA	Oki et al. (2009)
ACTH-GH	F/61	NA	NA	No	Acromegaly	GH, ACTH	Surgery	Recovery	Rasul et al. (2014)
ACTH-GH	F/78	NA	NA	NA	Acromegaly	GH	Surgery	Recovery	Rasul et al. (2014)
ACTH-GH	F/60	0.8	Left lobe of pituitary gland	No	Headaches and weight gain	GH	Surgery	Recovery	Roca et al. (2018)
ACTH-GH	F/30	1.4	Right side of butterfly saddle	Yes	Cushing’s syndrome	ACTH	Surgery	Recovery	Amir et al. (2022)
ACTH-PRL	F/42	NA	Extending from the suprasellar region to the sphenoid sinus and the right cavernous sinus	Yes	Hyperprolactinemia and Cushing’s syndrome	PRL, ACTH	Surgery	Recovery	Barausse et al. (2000)
FSH-PRL	F/29	0.7	Posterior pituitary	No	Irregular menstruation	FSH, PRL	Surgery	Recovery	Murata et al. (2003)
PRL-ACTH	M/31	1.5	Extending from the intrasellar region to the right cavernous sinus and eroding the sella	Yes	Hyperprolactinemia and Cushing’s syndrome	PRL, ACTH	Medicine	Recovery	T'sjoen et al. (2002)
TSH-PRL	F/52	3.0	NA	Yes	Thyrotoxicosis	TSH, PRL	Surgery	NA	Duello and Halmi (1977)
TSH-GH	M/13	4.0*4.5	With sphenoid bone and bilateral cavernous sinus infiltration, suprasellar extension, optic chiasma upward displacement, and compression of the left frontal lobe	Yes	Increased height, weight loss and eating more	TSH, GH	Surgery, Medicine and radiotherapy	Recurrence	Pereira et al. (2016)

ACTH, adrenocorticotropic hormone; PRL, prolactin releasing hormone; LH, luteinizing hormone; FSH, follicle-stimulating hormone; GH, growth hormone; TSH, thyroid stimulating hormone; F, female; M, male; NA, not applicable.

For decades, it was widely believed that pituitary adenomas were monoclonal (Beck-Peccoz et al., 1996; Dahia and Grossman, 1999; Levy and Lightman, 2003). There is much evidence to support the view that pituitary tumors are clonal lesions caused by defects in intrinsic pituitary cells, most of which are based on X chromosome inactivation (Chu et al., 2008; Sav et al., 2012). However, an increasing number of scholars have proposed that multiple synchronous adenomas are of polyclonal origin and that some sporadic tumors may also be polyclonal (Clayton and Farrell, 2001; Asa and Ezzat, 2002; Buch et al., 2002). PPA can potentially be explained also by heteroclonality (Herman et al., 1990; Levy and Lightman, 2003). In an earlier study based on nuclear DNA analysis by flow cytofluorimetry, biclonal cell lines were isolated in 10% of pituitary adenomas (Anniko and Tribukait, 1985). In more than half of the “recurrent” pituitary tumors with repeated analysis of loss of heterozygosity after further surgery, the loci involved in the original pattern of loss of heterozygosity were heterozygous again (Clayton et al., 2000). It also suggests that these pituitary tumors may be polyclonal. In addition, a growing number of studies have found that pituitary tumors can display intrinsic heterogeneity and cellular subpopulations with different biological, genetic and epigenetic properties (Sabatino et al., 2022). Tissue structure and microenvironment play a crucial role in tumors. Different cellular processes alter the structure and interactions within the gland, thereby shaping tumor growth. Tumor heterogeneity exists in many forms, ranging from somatic coding and non-coding alterations to epigenetic, transcriptomic and post-translational modifications (Caswell and Swanton, 2017; Mcgranahan and Swanton, 2017).

### Treatment

PPA is characterized by its aggressiveness and high postoperative recurrence rate (Steinbichler et al., 2018; Lodge et al., 2019; Lan and Behrens, 2023). Transsphenoidal microsurgical resection of mixed pituitary adenomas remains the preferred treatment for PPA (Swearingen, 2012; Ammirati et al., 2013). Surgical removal of tumors can reduce tissue pressure and hormone levels. However, it is not ideal for controlling tumor development for long-term remission. Radiotherapy is used only in patients whose tumor size or hormone levels are not sufficiently reduced by surgery or medication. Radiotherapy can be used as a postoperative adjuvant therapy. Importantly, the monitoring of pituitary function indefinitely after radiotherapy to detect anterior pituitary hypofunction is recommended. Approximately 50% of patients develop hypopituitarism within five years of radiation therapy (Loeffler and Shih, 2011; Ding et al., 2014; Molitch, 2017; Tritos and Miller, 2023). Wang et al. reported a case of GH-PRL adenoma in which programmed death ligand 1 (PD-L1) protein and CD8+ lymphocyte infiltrations were detected in tumor tissue. The tumor was aggressive and did not respond to bromocriptine therapy. GH and PRL levels did not improve after surgical resection and recurred postoperatively. The authors proposed the use of PD-1 inhibitors in combination with radiotherapy or temozolomide for the treatment of aggressive PPA (Wang et al., 2017) John et al. also found a higher incidence of significant PD-L1 in PIT-1-positive multihormone tumors than in other pituitary adenomas, and PD-L1 overexpression was very rare in ACTH and gonadotropin cells. The authors suggested that immunosuppressants may be a reasonable treatment for PIT-1 positive plurihormonal adenoma (Turchini et al., 2021).

## Conclusion

PPA is a relatively rare tumor that can secrete more than two hormones that differ in their biochemical composition, immunohistochemistry, and biological effects. We conducted a review of the literature to elucidate the clinical features of PPA and suggested the potential pathogenesis. We hypothesized that the pathogenesis of PPA is related to plurihormonal primordial stem cells, co-transcription factors, hormone co-expression, differential gene expression, and cell transdifferentiation. In the future, more basic and clinical studies are required to verify the pathogenesis and clinical features of PPA.

## Author contributions

YC: Conceptualization, Data curation, Formal analysis, Writing – original draft. SL: Investigation, Writing – review & editing. XZ: Funding acquisition, Project administration, Writing – review & editing. LR: Conceptualization, Methodology, Writing – review & editing. XL: Investigation, Writing – review & editing. XG: Resources, Validation, Visualization, Writing – review & editing. GW: Conceptualization, Funding acquisition, Project administration, Resources, Validation, Visualization, Writing – review & editing.
